# A Case Report on the Surgical Treatment of the Huge Inflammatory Pseudotumor in the AIDS Patient with Hemophilic

**DOI:** 10.1155/2011/798649

**Published:** 2011-09-05

**Authors:** Baochi Liu, Li Liu, Yanling Feng, Lei Li

**Affiliations:** Department of Surgery, Shanghai Public Health Clinical Center, Fudan University, 2901 Caolang Road, P.O. Box 201508, Jin Shan District, Shanghai, China

## Abstract

An HIV infected hemophilia patient with huge inflammatory pseudotumor was in severe ill condition. The operation of right hip joint amputation was performed on the patient with perioperative infusion of coagulation factor VIII and highly active antiretroviral therapy (HAART). The pathological found that Inflammatory cell infiltration, formation of folliculus lymphaticus, muscular fiber breakage, and fibrous tissue hyperplasy, necrosis in both soft tissue and bone were seen in inflammatory pseudotumour. The vital signs remained stable during the operation and patient's overall health condition improved significantly ten months after operation. With the infusion of coagulation factor VIII and HAART, HIV infected hemophilia patient can be safety operation and may get effective result.

## 1. Introduction

Hemophilic pseudotumors, first described in 1918 by Starker [1], are an encapsulated, chronic, slowly expanding hematoma with a severe coagulation disorder. Pseudotumors that occur in muscles with broad tendon insertions often progress to cause severe pressure erosion of adjacent bones. The number of reports in the literature about HIV-infected patients associated with Hemophilic pseudotumors remains limited. The patient with hemophilic inflammatory pseudotumor complicated by AIDS has very high risk for surgical treatment. Herein, we expose a case of HIV-positive patient who developed a rapidly growing hemophilic pseudotumors on the surgical treatment. 

## 2. Case Report

The patient, male, 20 years old, was diagnosed with Hemophilia. At the age of 2 due to continuous bleeding upon trauma. The patient was then treated with multiple infusion of coagulation factor VIII. In 2002, the patient was diagnosed with HIV infection combined with type B hepatitis and type C hepatitis virus infection. CD4^+^T cell was 70 cell/*μ*L in May 2002 and rose to 200 cell/*μ*L after the treatment using the highly active antiretroviral therapy (HAART). The right thigh swelling occurred in 2005 and was tentatively diagnosed as hematoma which increased gradually. On June 18, 2007, the patient was admitted to the Shanghai Public Health Clinical Center due to the swollen right thigh. The admission diagnoses were the following: (1) the right thigh hematoma, (2) AIDS, (3) hemophilia, (4) hepatitis C, and (5) hepatitis B. 

A group of surgical experts have been invited to consult on the case after the patient was admitted to the surgical ward. The consultation session concluded that the surgical indication is clear, but with extremely high risk for surgery. The initial treatment plan followed the conservative guideline. However, the hematoma increased gradually with oozing wound which exudated blood and body fluid daily and led to hypoalbuminemia with severe anemia. The patient was put on nutritional support such as albumin and the blood transfusion with daily dose of VIII factor 6000 u that maitin active VIII factor 40%–60%, human serum albumin 1 unit, infusion of the washed red blood cells 2 u every other day, plasma 200 mL in addition to the antivirus, antibiotics, and other drugs which added up to a daily healthcare cost of over 10 K RMB only to merely maintain the basic life-support with hemoglobin in the 15–40 g/L range. The heart failure and kidney failures occurred during the conservative treatment which required symptomatic treatment and intensive care. 

**Figure 1:**
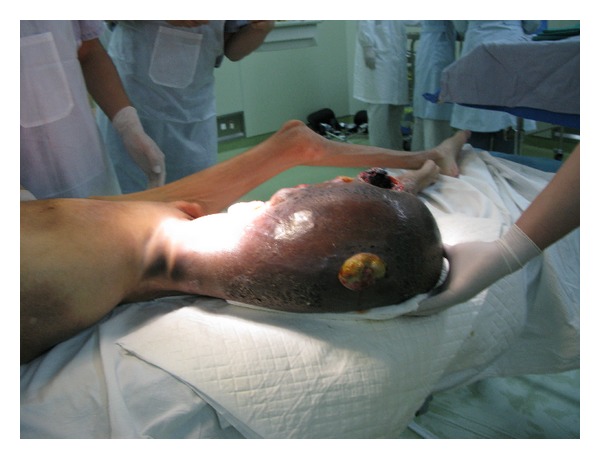
Inflammatory pseudotumor of the right lower limb.

**Figure 2:**
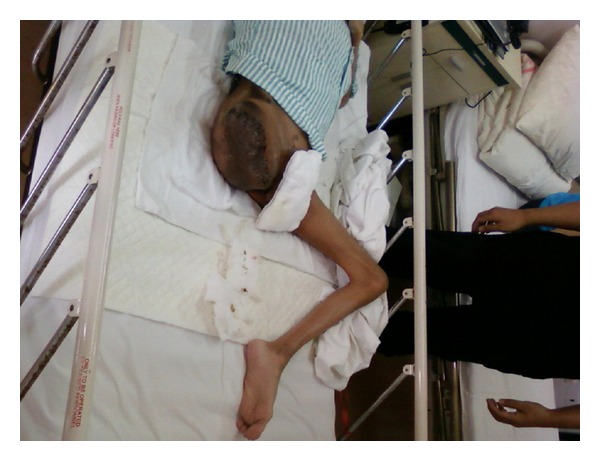
3 weeks after amputation, the cross-section wound first degree heal.

After careful preparation, the patient's CD4 level rose to 272 cell/*μ*L with the hemoglobin of 50.8 g/L and HIV-1 viral load dropped to below the detection level. On July 28, 2009, the joint relief operation was performed in patient's right hip under general anesthesia with intraoperative radial artery and central venous pressure monitoring (Figure 1). 2000 u of factor VIII infusion was applied before the operation followed by 4000 u of VIII factor during the operation. The operating room implemented standard disinfection and isolation procedures to prevent occupational exposure of the healthcare professionals to the HIV during the surgery. After the skin incision was made on the patient's right thigh, the truncated surface was carefully separated to cut off the blood vessels and minimize surgical bleeding. The right hip joint capsule was cut and the femoral neck had crisp fracture with bleeding on the femoral neck cross-section. The femoral head was removed from the acetabulum carefully and the drainage was placed followed by the suture closure of stump. The vital signs remained stable during the operation. The vital signs remained stable during the operation and 3 weeks after amputation, the cross-section wound first degree heal (Figure 2). Patient's overall health condition improved significantly ten months after operation.

**Pathological Findings**

**Figure 3:**
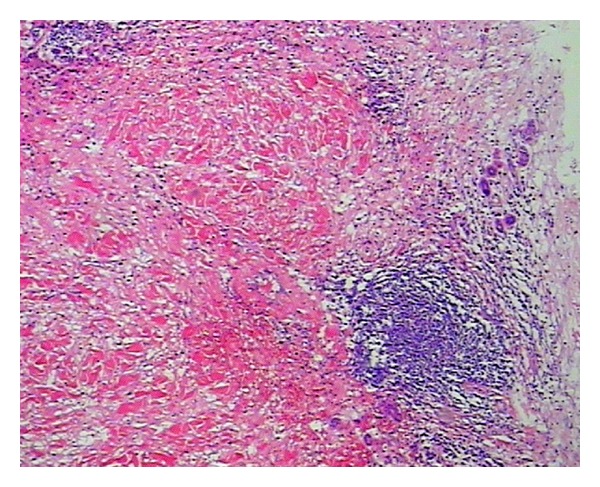
Inflammatory cell infiltration, formation of folliculus lymphaticus, muscular fiber breakage, and fibrous tissue hyperplasy were seen in inflammatory pseudotumour. HE ×40.

**Figure 4:**
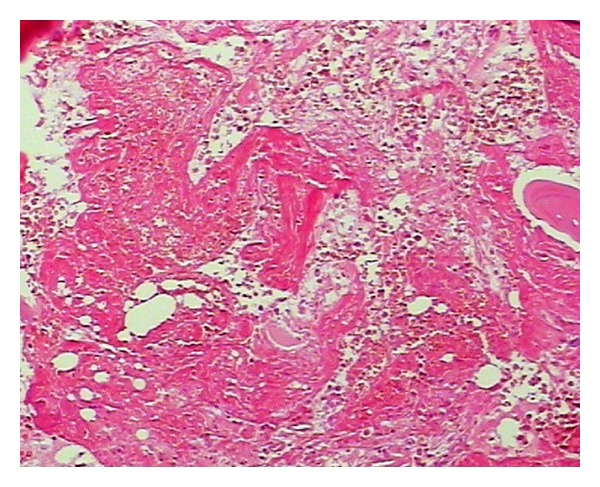
Necrosis in both soft tissue and bone, with haemorrhage in inflammatory pesudotumor. HE ×40.

The incision into the mass tissue, the intersection is taupe color with bone-like tissue with partial decalcification. The hemorrhagic necrosis of subcutaneous tissue and muscle was observed along with the neutrophils, lymphocytes and macrophages infiltration, and the femoral head necrosis (Figures 3 and 4). The review checkup 6 days post-op showed a hemoglobin 92 g/L accompanied by a significant improvement of the patient overall condition with all vital signs stable. The daily dose of the VIII factor was gradually reduced, and the surgical wound had a first degree heal with complete removal of stitches 3 weeks after surgery.

## 3. Discussion

Hemophilia is a group of inherited blood coagulation disorders diseases with bleeding symptoms being the main manifestations of this disease [2, 3]. Hemophilic arthritis is mainly caused by the repeated bleeding inside the joints. The epiphysis and metaphysis bleeding can cause the deformation and collapse of the epiphyseal and may result in aseptic necrosis which is relatively rare. The inflammatory pseudotumor of hemophilia caused the repeated bleeding inside the bone or the cartilage which lead to the bone absorption or the cystic degeneration. The bone marrow, joint capsule and the joint may get damaged or develop necrosis due to the mechanical and chemical stimulation by the cellulose, hemosiderin, and other substances in the blood. The rising intraosseous pressure may cause osteolytic bone destruction and the swelling damage. In some instances, the extreme swelling led to the breakthrough of the cortical bone. The pseudotumor often associated with soft tissue swelling and periosteal reaction. The hyperplasia of the periosteum may risk further damage. The pseudotumor occurred in the long bones can easily cause pathological bone fractures, which easily get misdiagnosed as the bone tumor if patient's clinical history of easy bleeding was neglected. Hemophilic pseudotumor has X-ray characteristics of both benign and malignant bone tumors, such as bone destruction, periosteal reaction, and new bone formation [4–7]. Therefore, once the patient is admitted, the doctor should check details of the patient clinical history and combine then with other checkup data to make the correct diagnosis.

**Figure 5:**
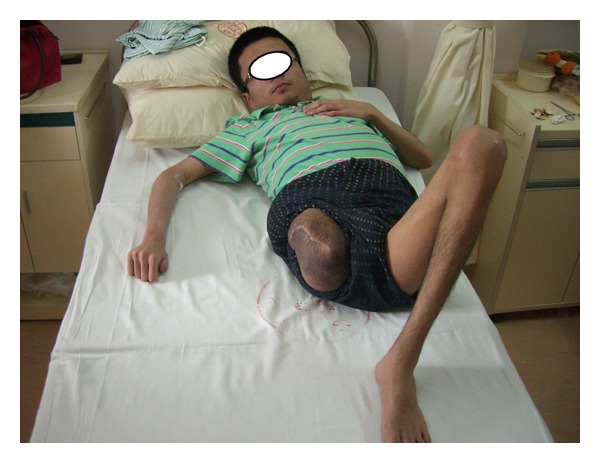
Ten months after operation patient's overall health condition improved significantly.

The patient in this case study started with the swelling on the right thigh above the knee, initially diagnosed as hematoma. However, after infusion of coagulation factor VIII and other treatment, the hematoma did not get absorbed, but continued to grow. After the development of a huge mass of the thigh, only amputation can remove the lesion. Initially the patient family did not agree to amputation. But the general support treatment cannot control the development of the inflammatory pseudotumor. When the pseudotumor became highly swollen with ulceration, high daily doses of blood clotting factor VIII, plasma, albumin, red blood cells, and so forth were required to merely sustain life. By the time the patient family agreed to the amputation, the patient had suffered from severe anemia and hypoalbuminemia, combined with HIV, hepatitis C, and hepatitis B virus infection with extremely high risk for surgery. Shanghai Public Health Clinical Center (SPHCC) is the diagnosis and treatment center and the designated hospital for the AIDS-related diseases. The SPHCC is responsible for treatment of all the HIV-infected patients, which is an important task. The patient in this case study has complications of AIDS hemophilia with giant inflammatory pseudotumor in addition to the hepatitis B and hepatitis C severe anemia, hypoalbuminemia, and immune dysfunction. Any additional slight health issue will be potentially life threatening and the surgery is of extremely high risk [8–13]. However, the surgical treatment is the only hope for an effective treatment in this case which justified the undertaking of the high risk surgery. The key is to control the surgical bleeding. Since the inflammatory pseudotumor is huge with the upper edge near the hip joint, we had to disarticulate close to the edge of inflammatory pseudotumor in order to ensure there are sufficient skin flap to wrap around the stump. After the gentle skin incision with scalpel, there was obvious subcutaneous leakage. The suction apparatus was used to suck the wound exudates and push the subcutaneous tissue to reveal the root of each small blood vessels for accurate ligation or electrocoagulation to stop bleeding. The stock artery was separated with a double ligation and cut off of the femoral vein to reveal a clear surgical filed with very little bleeding. When the hip joint capsule was broken away, the femoral neck had a crisp fracture due to the femoral head necrosis. The femoral neck cross-section continuously oozed blood which increased the surgical difficulty. The femoral head was carefully removed from the acetabulum with the complete surgical ligation or electrocoagulation to stop bleeding wound. Finally the drainage tube was placed followed by the suture closure of stump. The total blood loss during the surgery was less than 300 mL. The Preoperative infusion of 2000 u factor VIII combined with the intraoperative infusion of 4000 u of factor VIII ensured that the hemophilic patient had normal coagulation function similar to the nonhemophilic patient and that there was no obvious oozing wound during the surgery. The daily infusion of 6000 u factor VIII continued after operation. About 300 mL of pale red liquid drained from the wound drainage tube on the day of the surgery. The drainage from the drainage tube reduced gradually with the reducing edema of the right hip of the patient caused by the inflammatory pseudotumor. The drainage tube was removed 7 days after the surgery and the stitches around the stump was removed 3 weeks after amputation with the first degree wound heal, (see Figures 1 and 2). Due to the surgical removal of the pseudotumor, the patient's overall health condition improved significantly ten months after operation (Figure 5).
